# KDM4C Contributes to Trophoblast-like Stem Cell Conversion from Porcine-Induced Pluripotent Stem Cells (piPSCs) via Regulating CDX2

**DOI:** 10.3390/ijms23147586

**Published:** 2022-07-08

**Authors:** Shuai Yu, Qiaoyan Shen, Rui Zhang, Xiaolong Wu, Juqing Zhang, Wenxu Zhao, Xiaojie Wu, Na Li, Sha Peng, Shiqiang Zhang, Fan Yang, Jinlian Hua

**Affiliations:** 1 College of Veterinary Medicine, Shaanxi Centre of Stem Cells Engineering & Technology, Northwest A&F University, No. 22 Xinong Road, Yangling District, Xianyang 712100, China; 2017060234@nwafu.edu.cn (S.Y.); sqy@nwafuedu.cn (Q.S.); zrui@nwafu.edu.cn (R.Z.); 2018050593@nwafu.edu.cn (X.W.); 2013015382@nwafu.edu.cn (J.Z.); zhaowx@nwafu.edu.cn (W.Z.); wuxiaojie@nwafu.edu.cn (X.W.); lina2017@nwsuaf.edu.cn (N.L.); pengshacxh@nwafu.edu.cn (S.P.); shiqiangzhang@nwafu.edu.cn (S.Z.); fanyang@nwafu.edu.cn (F.Y.); 2 College of Veterinary Medicine, Yangzhou University, Yangzhou 225009, China

**Keywords:** porcine-induced pluripotent stem cells (piPSCs), trophoblast stem cells (TSCs), epigenetic modification, KDM4C, CDX2

## Abstract

Studies on ESRRB-regulating porcine-induced pluripotent stem cells (piPSCs) converted to trophoblast-like stem cells (TLSCs) contribute to the understanding of early embryo development. However, the epigenetic modification regulation network during the conversion is poorly understood. Here, the global change in histone H3 Lysine 4, 9, 27, 36 methylation and Lysine 27 acetylation was investigated in piPSCs and TLSCs. We found a high modification profile of H3K36me2 in TLSCs compared to that of piPSCs, whereas the profiles of other modifications remained constant. KDM4C, a H3K36me3/2 demethylase, whose gene body region was combined with ESRRB, was upregulated in TLSCs. Moreover, KDM4 inhibitor supplementation rescued the AP-negative phenotype observed in TLSCs, confirming that KDM4C could regulate the pluripotency of TLSCs. Subsequently, KDM4C replenishment results show the significantly repressed proliferation and AP-positive staining of TLSCs. The expressions of CDX2 and KRT8 were also upregulated after KDM4C overexpression. In summary, these results show that KDM4C replaced the function of ESRRB. These findings reveal the unique and crucial role of KDM4C-mediated epigenetic chromatin modifications in determination of piPSCs’ fate and expand the understanding of the connection between piPSCs and TSCs.

## 1. Introduction

After fertilization, a totipotent zygote undergoes nuclear reprogramming and chromatin remodeling, and then forms blastocyst, which consists of inner cell mass and trophoblasts [1]. Fetal abortion is caused by placenta-related complications, such as the abnormal differentiation of trophoblast lineages in mammalian pregnancy [2]. Nevertheless, obtaining accurate in vivo data to thoroughly investigate trophoblast development in the uterus after implantation is difficult owing to technical difficulties and ethical restrictions. A novel approach to study early trophoblast development is to employ induced pluripotent stem cells (iPSCs). Thus, how to build a system from iPSCs to trophoblasts has become a hot issue in current mammalian research. Naïve human iPSCs can directly give rise to trophoblast-like stem cells (TLSCs) and undergo further differentiation into extravillous trophoblasts and syncytiotrophoblasts [3]. Recently, Io et al. established an in vitro model of the trophoblast lineage from human iPSCs [4]. We also found that Estrogen-Related Receptor Beta (ESRRB) overexpression in pig iPSCs (piPSCs) could cause the trans-differentiation of piPSCs into TLSCs [5]. This result reflects the powerful function of the transcription factor ESRRB. However, gene expression depends on various genetic and epigenetic chromatin modifications, especially histone post-translational modifications (PTMs) during this process [6]. Thus, the study of PTMs is important for a wide range of scientific and industrial processes in piPSCs.

PTMs play essential roles in gene regulation and chromatin packaging in eukaryotes [7]. The dynamical alteration of PTMs would be choreographed to regulate the cellular process, such as transcription, replication, recombination and DNA repair [8]. In particular, histone methylation significantly participates in controlling cell fate and differentiation [9]. One of the essential histone modifications, namely, histone H3 lysine-36 methylation (H3K36me), is modified by the methylases and demethylases onto the H3 tail and is conserved from yeast into eukaryotes [10]. Recent studies predicted that H3K36me is a silencing mark associated with cryptic transcription within gene bodies and DNA repair [11]. As an H3K9me3 and H3K36me3/2 demethylase, lysine demethylase 4c (Kdm4c) has been reported to be an oncogene in cancers that regulates cell proliferation by activating the genes involved in cell signaling, cell cycle and translation [12,13]. In addition, Kdm4c also impairs cleavage-stage development and embryonic stem cells’ (ESCs) integrity [14]. Kdm4c could target the H3K4me3-rich gene promoter regions in ESCs through its core Tudor domains [15]. Kdm4c cooperates with PRC2 and Nanog in transcriptional activation and repression, respectively [16,17]. Thus, we predicted that Kdm4c could act as a crucial factor in the pluripotency and differentiation of pluripotent stem cells.

The change in cell state is regulated by epigenetics to some extent. Thus, we conjectured the histone modifications might be regulated during the conversion to TLSCs from piPSCs. In this study, we detected the patterns of histone modifications during the conversion of piPSCs into TLSCs, and found that H3K36me2 was the most intense and variable parameter during the process. Moreover, H3K36me3/2 demethylase and KDM4C could activate CDX2 expression. In summary, our data demonstrate a novel role of ESRRB in TLSC induction by targeting KDM4C.

## 2. Results

### 2.1. Characterization of the Histone Modifications in the E-piPSCs

**Figure 1:**
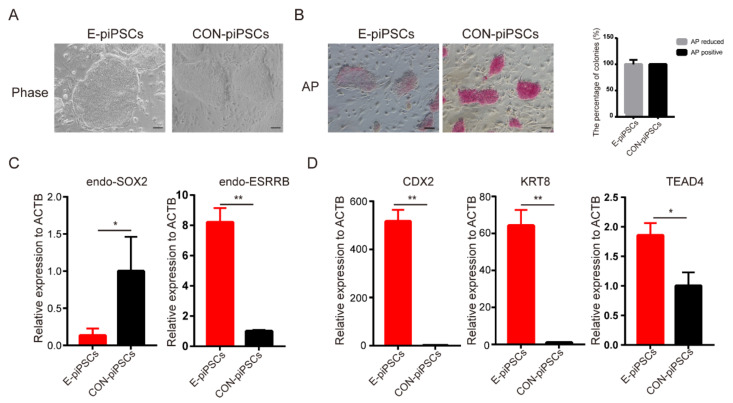
Characterization of E-piPSCs and CON-piPSCs. (**A**) Representative image of bright field of the CON- and ESRRB-overexpressing piPSCs (E-piPSCs); the scale bar represents 100 μm. (**B**) AP staining and qualities analysis of the CON and E-piPSCs; the scale bar represents 100 μm. (**C**,**D**) qRT-PCR analysis of the expression levels of pluripotent (*SOX2*, *ESRRB*) and trophoblast (*CDX2*, *KRT8* and *TEAD4*) markers in the CON- and E-piPSCs. * represents *p* < 0.05 and ** represents *p* < 0.001.

ESRRB played a critical role in pluripotent stem cells and trophoblast maintenance. In order to reveal this function of ESRRB in piPSCs, a piPSCs line with the overexpression of ESRRB (named as E-piPSCs) was established. When compared with CON-piPSCs, the E-piPSCs showed scattered and flat colonies (Figure 1A). AP staining analysis showed that the AP-positive rate was significantly reduced in E-piPSCs compared with CON-piPSCs, which we predicted would show reduced pluripotency due to ESRRB overexpression (Figure 1B). The mRNA level of the pluripotent stem cell marker, *endogenous SOX2* (*endo-SOX2*), was significantly reduced in E-piPSCs compared to that in CON-piPSCs (Figure 1C). These results indicate that the state of piPSCs was changed by ESRRB. To determine the character of these differentiated cells, we inspected early lineage markers. Interestingly, we found that the expressions of TSCs markers, such as *CDX2*, *KRT8* and *TEAD4*, could be activated by ESRRB in the piPSCs, which suggested that the cells were in the TLSC state (Figure 1D). Moreover, the *endogenous ESRRBs* (*endo-ESRRBs*) were upregulated in E-piPSCs when compared with that in CON piPSCs (Figure 1C). In a word, ESRRB could facilitate the conversion of TLSCs from piPSCs.

**Figure 2:**
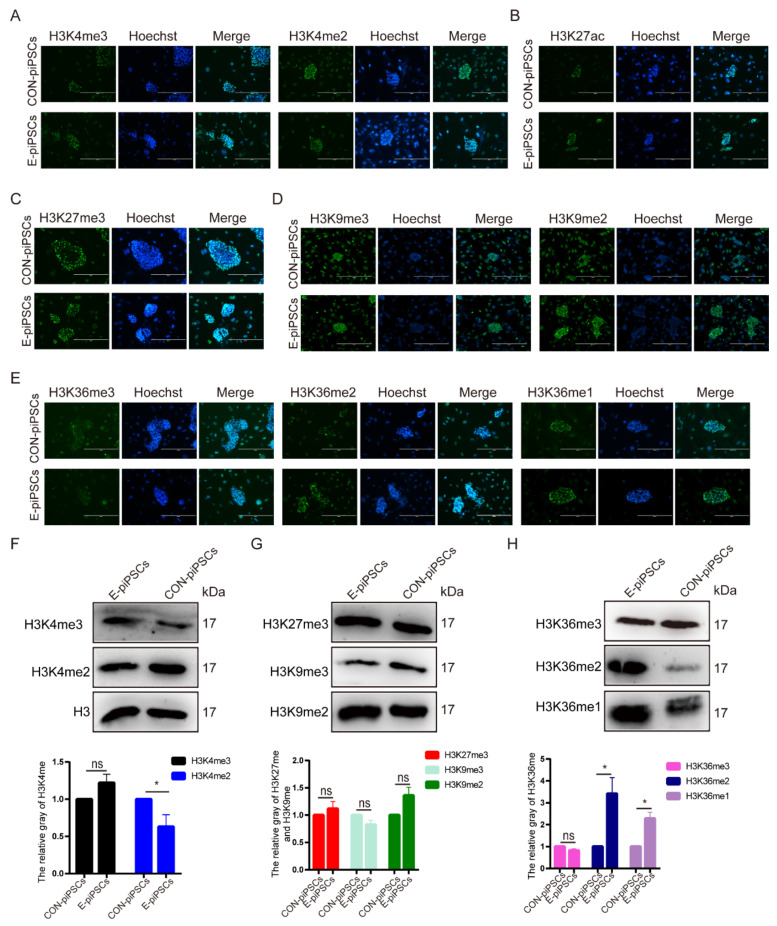
Characterization of the histone modifications in the CON- and E-piPSCs. (**A**) Immunofluorescence of the H3K4me2/3 and H3K27ac levels in the CON- and E-piPSCs; the scale bar represents 400 μm. (**B**) Immunofluorescence of the H3K27ac levels in the CON- and E-piPSCs; the scale bar represents 400 μm. (**C**) Immunofluorescence of the H3K9me2/3 levels in the CON- and E-piPSCs; the scale bar represents 400 μm. (**D**) Immunofluorescence of the H3K27me3 levels in the CON- and E-piPSCs; the scale bar represents 400 μm. (**E**) Immunofluorescence of the H3K36me1/2/3 levels in the CON- and E-piPSCs; the scale bar represents 400 μm. (**F**–**H**) Western blot analysis of the H3K4me2/3, H3K9me2/3, H3K27me3 and H3K36me1/2/3 levels in the CON- and E-piPSCs. The histogram data included quantification of blots, and H3 was a loading control. ns represents *p* > 0.05, * represents *p* < 0.05.

Epigenetic histone modifications are known to have a major impact on pluripotent maintenance. Then, we tried to investigate possible effects on histone modifications during the conversion of TLSCs from piPSCs. At first, we detected the levels of Histone H3 lysine-4 methylation (H3K4me) (Figure 2A), Histone H3 lysine-27 acetylation (H3K27ac) (Figure 2B), Histone H3 lysine-27 methylation (H3K27me) (Figure 2C) and Histone H3 lysine-9 methylation (H3K9me) (Figure 2D). There was no significant difference in enrichment as analyzed by immunofluorescence in E-piPSCs compared to CON-piPSCs. Then, we tried to check the levels of Histone H3 lysine-36 methylation (H3K36me), whose function is unknown. As shown in Figure 2E, we found significant enrichment in H3K36me2/1 levels in E-piPSCs compared to those of CON-piPSCs accompanied by a significant reduction in H3K36me3 levels in E-piPSCs compared to that of CON-piPSCs. We used the primary antibody-free group as a negative control, and found no staining in the visual field (Figure S1A).

In order to further confirm this finding, Western blot analysis was carried out to detect the levels of histone modifications in the E-piPSCs and CON-piPSCs. Consistent with immunofluorescence, there was a slight increase in the level of H3K4me3 in E-piPSCs compared to that of CON-piPSCs, and the level of H3K4me2 was slightly reduced in E-piPSCs compared that of CON-piPSCs. Moreover, there were no significant differences in relative enrichment in H3K27me3 and H3K9me3/2 in E- and CON-piPSCs (Figure 2G). In addition, Western blot analysis showed the significant enrichment in H3K36me2/1 levels in E-piPSCs compared to those of CON-piPSCs (Figure 2H). These results predict that the enrichment in H3K36me2 modification establishment might play a role in the conversion of TLSCs from piPSCs.

### 2.2. Characterization of the Histone Demethylase KDM4C

**Figure 3:**
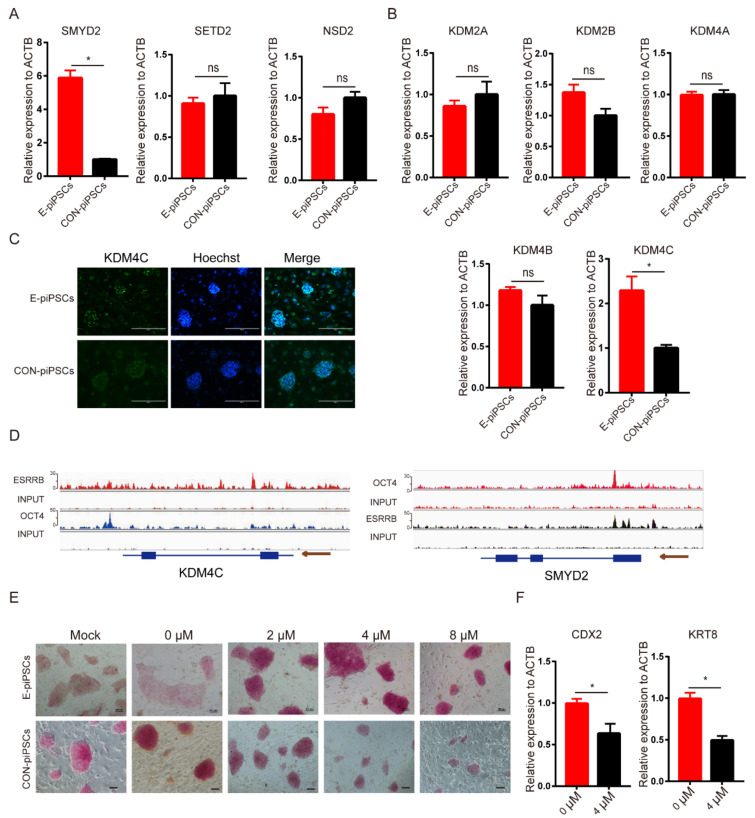
KDM4C is the core factor in the E-piPSCs. (**A**) qRT-PCR analysis of H3K36me methylases in CON- and E-piPSCs; * represents *p* < 0.05 and ns represents *p* > 0.05. (**B**) qRT-PCR analysis of H3K36me demethylases in CON- and E-piPSCs; * represents *p* < 0.05. (**C**) The immunofluorescence staining of KDM4C in CON- and E-piPSCs; the scale bar represents 200 μm. (**D**) ChIP-seq analysis of ESRRB marks at KDM4C and SMYD2 using IGV. The size of the peak represents the degree of enrichment. (**E**) Representative image of AP-stained colonies after adding ML324 in CON- and E-piPSCs; the scale bar represents 100 μm. (**F**) qRT-PCR analysis of CDX2 and KRT8 in E-piPSCs after adding 4 μM ML324; * represents *p* < 0.05.

Our results indicate that H3K36me2 might be affected by ESRRB; furthermore, ESRRB has not been reported to regulate H3K36 methylation and demethylation directly. We detected the level of H3K36me2 methylases and demethylases, which could regulate the enrichment in H3K36me2 levels. As shown in Figure 3A, the expression level of H3K36me1 methylase (*SMYD2*) was upregulated in E-piPSCs when compared with CON-piPSCs, and the expression of H3K36me3 methylase (*SETD2*) and H3K36me2 methylase (*NSD2*) was not significantly changed. Moreover, the expression levels of H3K36me3/2 demethylase and KDM4C were upregulated in E-piPSCs when compared with CON-piPSCs, but other H3K36me3/2 demethylases (*KDM4A* and *KDM4B*), and the H3K36me2/1 demethylases (*KDM2A*, *KDM2B*), were not significantly changed (Figure 3B). The immunofluorescence analysis also predicted that the level of KDM4C was upregulated in E-piPSCs compared to CON-piPSCs (Figure 3C and Figure S2A). The results indicate that KDM4C and SMYD2 might be the core factors which affect the enrichment in H3K36me2 levels. 

Thus, the ChIP-seq analysis was used to detect whether KDM4C and SMYD2 were the direct downstream target genes of ESRRB. The anti-FLAG ESRRB piPSCs were used to conduct ChIP-Seq, and the CON-piPSCs were used as a negative control. The ChIP-seq quality control is shown in Figure S3. Further analysis to determine whether ESRRB ChIP-seq data were actually targeted by the DNA of KDM4C was carried out. The ChIP results show that the enhancer regions upstream of KDM4C were directly bound by OCT4 and ESRRB, and the enhancer and promoter regions of SMYD2 were directly bound by ESRRB (Figure 3D). The result demonstrates that ESRRB directly regulates the expression of KDM4C and SMYD2. In summary, ESRRB could activate the expressions of KDM4C and SMYD2 (Figure 3D). 

Then, we tried to explore whether KDM4C could affect the conversion of TLSCs from piPSCs. We chose the KDM4 inhibitor, ML324, to disrupt KDM4C function. The concentrations of ML324 on piPSCs were determined via AP staining. The rate of AP-positive colonies increased with concentrations of ML324 between 2 and 4 μM in E-piPSCs compared to when ML324 was not used (Figure 3E). However, the colony size of E-piPSCs decreased when treated with a high concentration (8 μM) of ML324 (Figure 3E). Moreover, the colony size of CON-piPSCs also decreased with the addition of ML324 (Figure S2B). We speculated that the high concentration of ML324 might have resulted in fewer and smaller colonies and used EdU staining to verify it (Figure S2C). Then, we chose 4 μM ML324 to treat the E-piPSCs so that we could not only achieve the highest AP-positive colony ratio but also maintain cell proliferation normally. qRT-PCR results show that the expressions of CDX2 and KRT8 were significantly reduced in the 4 μM group compared to those of the 0 μM group (Figure 3F). These results show that KDM4C played a critical role in the pluripotency and TLSCs’ conversion of E-piPSCs, but a higher concentration of KDM4C inhibitor could also affect cell proliferation.

### 2.3. CDX2 Is the Key Target of KDM4C

**Figure 4:**
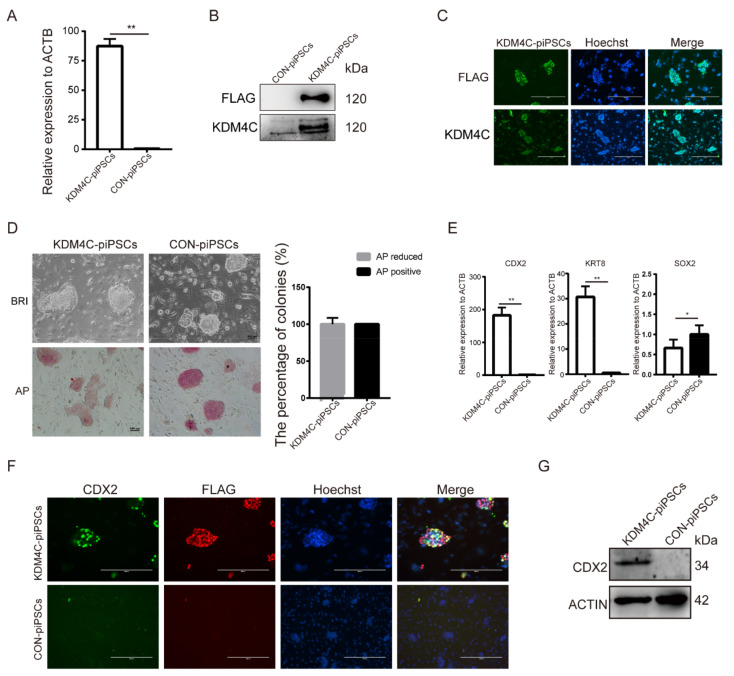
KDM4C activated the expression of CDX2. (**A**) qRT-PCR analysis of KDM4C in CON- and KDM4C-overexpressing piPSCs (KDM4C-piPSCs); ** represents *p* < 0.001. (**B**) Western blot analysis of the KDM4C and FLAG expression level in the CON- and KDM4C-piPSCs; (**C**) immunofluorescence analysis of KDM4C and FLAG in the KDM4C-piPSCs; the scale bar represents 400 μm. (**D**) Representative image of bright-field and AP-stained colonies in the CON and KDM4C-piPSCs; the scale bar represents 100 μm. (**E**) qRT-PCR analysis of CDX2, KRT8 and SOX2 in E-piPSCs in the CON and KDM4C-piPSCs; ** represents *p* < 0.001, * represents *p* < 0.05. (**F**) Immunofluorescence analysis of CDX2 and FLAG level in the CON- and KDM4C-piPSCs; the scale bar represents 200 μm. (**G**) Western blot analysis of the CDX2 level in the CON- and KDM4C-piPSC.

To investigate this potential role of KDM4C in piPSCs, we generated KDM4C-overexpressing piPSCs using lentivirus vector (marked as KDM4C-piPSCs) in which KDM4C expression was enhanced. qRT-PCR and Western blot analyses revealed that the expression of KDM4C was significantly stronger in KDM4C-piPSCs than that of CON-piPSCs (Figure 4A,B). Immunofluorescence showed that the KDM4C was observed in the nucleus of KDM4C-piPSCs, in accordance with its role as a transcription factor (Figure 4C). Notably, KDM4C-piPSCs formed flat colonies, and showed attenuated or even negative AP stains (Figure 4D). The qRT-PCR results show that the expression levels of CDX2 and KRT8 were upregulated in the KDM4C-piPSCs when compared with CON-piPSCs, but the expression of SOX2 showed the opposite (Figure 4E). Immunofluorescence and Western blot analyses revealed that overexpression of KDM4C resulted in upregulation of CDX2 (Figure 4F,G).

**Figure 5:**
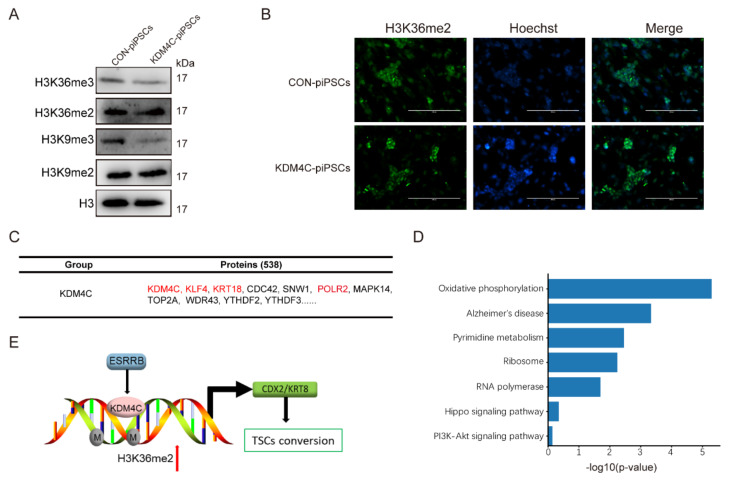
The expression levels of H3K36me1/2/3 in CON− and KDM4C−piPSCs. (**A**) Western blot analysis of H3K36me2/3 and H3K9me2/3 in CON- and KDM4C-piPSCs; (**B**) the immunofluorescence staining of H3K36me2 in CON- and KDM4C-piPSCs; the scale bar represents 400 μm. (**C**) The table lists FLAG-enriched KDM4C-associated proteins in KDM4C-piPSCs detected by immunoprecipitates–MS analysis; the CON-piPSCs was used as a control. (**D**) The KEGG enrichment in KDM4C-associated proteins in KDM4C-piPSCs; (**E**) model for action of ESRRB, KDM4C and CDX2 during pluripotency conversion into TLSCs.

In this process, we found that there were no other external factors except for the expression of KDM4C; then, in order to detect the function of histone modification, we detected the level of both activating and silencing histone modifications, which showed the significant reduction in H3K36me3 and H3K9me3 in KDM4C-piPSCs compared to CON-piPSCs (Figure 5A and Figure S4A), and we also found the enrichment in H3K36me2 in KDM4C-piPSCs compared to that of CON-piPSCs (Figure 5A,B and Figure S4B). In order to exclude the interference of H3K9me3, we also generated KDM3A, an H3K9me3/2-demethylase-overexpressing piPSC, using a lentivirus vector. However, in KDM3A-piPSCs, there was no expression of CDX2. These results show that KDM4C could regulate the expression of CDX2 by modifying H3K36me2, and the ESRRB could also regulate the expression of KDM4C.

To further understand the molecular mechanism of KDM4C and CDX2, we applied immunoprecipitates–MS to directly investigate the proteins related to KDM4C (Figure 5C). The mass spectrometry results show that there were 538 proteins that were directly bound by KDM4C, such as KLF4, KRT18, CDC42, SNW1, POLR2 and so on, but not CDX2 or KRT8. The specific KDM4C-interacting proteins were enriched with functions, including the positive regulation of Alzheimer’s disease, ribosomes, RNA polymerase, the Hippo signaling pathway and PI3K-Akt signaling pathway (Figure 5D).

## 3. Discussion

In our recent study, we found that ESRRB could trigger piPSC differentiation into TLSCs through activating CDX2 and KRT8 expression [5]. However, the mechanisms of histone modifications underlying the conversion process are yet to be established. In this study, we found that the H3K36me2 level considerably increased in E-piPSCs compared to in CON-piPSCs, and the histone demethylase, KDM4C, played an important role in this process. Moreover, overexpressing KDM4C could activate CDX2 expression and affect piPSC proliferation. Our findings provide an enhanced framework for understanding how H3K36me2 landscapes are established and maintained in the conversion of TLSCs from piPSCs (Figure 5E). Moreover, we found that KDM4C provides a basis for the connection between histone methylation and stem cell transformation.

Epigenetics refers to the stable and inheritable changes in gene expression without alterations in DNA sequences, and is crucially involved in gene regulation, cellular differentiation, gene imprinting, X-chromosome inactivation and other cellular processes [18]. Previous studies evaluating H3K36me2 observed inconsistent results for recruiting DNMT3A and shaping the intergenic DNA methylation landscape [19]. Moreover, H3K36me2 could demarcate PRC2-mediated H3K27me2 and H3K27me3 domains in ESCs [20,21]. However, the functions of H3K36me2 in piPSCs and TSCs are unknown. In this study, we found an increase in H3K36me2 levels, but not in other histone modifications in TLSCs compared with piPSCs. Then, we tried to detect the detailed regulation mechanism of H3K36me2.

At first, we detected the expression of histone-modifying enzymes in E-piPSCs and found that KDM4C could be a candidate. Unlike many other histone-modifying enzymes, KDM4C demethylates H3K36me3/2 and H3K9me3 at the same time [22]. However, the function of KDM4C is always hard to determine. Recent studies showed that KDM4C, in cooperation with PRMT1, remodeled epigenetic programs and deregulated transcriptional patterns by removing the repressive effect of H3K9me3 on HOXA9, and could maintain the survival of AML [13]. In ESCs, KDM4C is required for successful multi-lineage differentiation, as assessed by EB formation. In the absence of KDM4C, EBs were smaller and ineffective in inducing the expression of differentiation-associated genes [23]. Moreover, KDM4C could participate in the regulatory function of the tissue-specific enhancers during ESCs priming for differentiation. In this study, we found that KDM4C overexpression could activate CDX2 expression. The immunoprecipitates–MS results show that KDM4C might interact with SNW1 and POLR2 to participate in cell transcription. Moreover, KDM4C might participate in the stem cell function with KLF4. However, KDM4C could not interact with CDX2 and KRT8, and it was predicted that KDM4C is just an upstream gene of CDX2 and KRT8, and not a partner. Remarkably, ESRRB was similarly enriched at active and pre-marked (poised) enhancers of KDM4C, suggesting that KDM4C could act as a downstream target gene of ESRRB to regulate the pluripotency and differentiation of piPSCs.

## 4. Materials and Methods

### 4.1. piPSC Culture

The ESRRB-overexpressing piPSCs (E-piPSCs) and CON-piPSCs were cultured on Mitomycin C-treated mouse embryonic fibroblast feeder cells and cultured in DMEM medium supplemented with 15% FBS (VISTECH, Auckland, New Zealand), 4 μg/mL doxycycline (DOX, Sigma Aldrich, St. Louis, MO, USA), 0.1 mM NEAA (Gibco, Waltham, MA, USA), 1 mM L-glutaMAX (Gibco, Waltham, MA, USA), 0.1 mM β-mercaptoethanol (Sigma Aldrich, St. Louis, MO, USA), 10 ng/mL LIF (Sino Biological, Beijing, China), 10 ng/mL bFGF (Sino Biological), 3 µM CHIR99021 (MCE, South Bend, IN, USA) and 2 µM SB431542 (Selleck, Houston, TX, USA) [24]. The cells were passaged using TrypLE™ Select into a single cell at 2 × 10^4^ cells per 12-well plate every 6 days.

### 4.2. RNA Extraction and Quantitative Real-Time Polymerase Chain Reaction (qRT-PCR) Analysis

Total RNA from each of the E- and CON-piPSCs was extracted by using Trizol reagent (TaKaRa, Dalian, China) according to the manufacturer’s protocol. The cDNA was synthesized by reverse transcription PCR using the PrimeSript™ RT reagent Kit (Tiangen, Beijing, China) according to the manufacturer’s protocol. The qRT-PCR reaction system included, in volume: 10 μL of SYBR^®^ Premix Ex Taq II (Tiangen) (2×), 1 μL of cDNA, and 1 μL of PCR Forward/Reverse Primer (10 μmol/L), and RNase-free water was added to achieve a total volume of 20 μL. The specific primers of genes used in this study are shown in Table 1. Relative mRNA expression levels of these genes for each treatment were normalized by the geometric mean of β-actin and were computed by the 2^−ΔΔCT^ method [25]. β-ACTIN was used as an endogenous loading control.

### 4.3. Alkaline Phosphatase (AP) Staining

The pluripotency of E- and CON-piPSCs was determined by AP staining solution (AST Fast Red TR and α-Naphthol AS-MX Phosphate (Sigma Aldrich)) according to the manufacturer’s instruction. In brief, the piPSCs were incubated with 4% paraformaldehyde (Sangon Biotech, Shanghai, China) for 15 min and then washed twice with PBS. Afterwards, the cells were incubated with 0.1 M Tris Buffer consisting of 1.0 mg/mL Fast Red TR and 0.4 mg/mL Naphthol AS-MX for 20 min. Then, the pluripotency of piPSCs was detected by the color intensity of colonies using a phase-contrast microscope (Nikon, Tokyo, Japan) [26].

### 4.4. Immunofluorescence 

The immunofluorescence analyses were used to evaluate the characteristics of piPSCs. Firstly, the cells were incubated with 4% paraformaldehyde and 0.05% Triton X-100 for 15 min, respectively. After that, the cells were blocked by 10% FBS solution for 60 min. Subsequently, the piPSCs were incubated with specific primary antibodies at 4 °C for 12–16 h. Then, the cells were incubated for 1 h with appropriate secondary antibodies (1:500, ZSGB-BIO, Beijing, China), and labeled with Hoechst33342 (1:1000; Beyotime, Shanghai, China) for 5 min at 37 °C. Finally, the immunofluorescence results were captured by the EVOS M5000 fluorescence microscope (Thermo, Waltham, MA, USA). The specific primary antibodies used in this study included: H3K4me3 antibody (Rabbit; Active Motif, Carlsbad, CA, USA); H3K4me2 antibody (Rabbit; Active Motif); H3K9me3 antibody (Rabbit; Active Motif); H3K9me2 antibody Rabbit; Active Motif); H3K27ac antibody (Rabbit; Active Motif); H3K27me3 antibody (Rabbit; Active Motif); H3K36me1/2/3 antibody (Rabbit; Active Motif); CDX2 (Rabbit; Bioss, Beijing, China); FLAG (Mouse; Sigma, St. Louis, MO, USA); KDM4C (Rabbit; Sangon Biotech, Shanghai, China) [27].

### 4.5. Western Blot Analysis

The Western blot analyses were used to evaluate the characteristics of piPSCs. Firstly, the piPSCs precipitation was lysed by cold RIPA buffer (Beyotime, Shanghai, China) for 30 min, and then heated at 100 °C with loading buffer for 10 min. The extracted proteins lysates were analyzed with SDS-PAGE. The different proteins were separated by 8–12% SDS-PAGE and transferred electrophoretically onto polyvinylidene difluoride (PVDF) membranes by Trans-Blot SD Cell and Systems (Bio-Rad, Hercules, CA, USA) for 50 min at 15 V. Blocked by 5% non-fat milk in TBST buffer, the proteins attached PVDF membranes were incubated with the primary antibodies, and HRP-conjugated secondary antibody (Life Technology, Carlsbad, CA, USA). The specific primary antibodies used in this study included: H3K4me3 antibody (Rabbit; Active Motif; USA); H3K4me2 antibody (Rabbit; Active Motif); H3K9me3 antibody (Rabbit; Active Motif); H3K9me2 antibody Rabbit; Active Motif); H3K27me3 antibody (Rabbit; Active Motif); H3K36me1/2/3 antibody (Rabbit; Active Motif); H3 antibody (Rabbit; Active Motif); CDX2 (Rabbit; Bioss, China); FLAG (Mouse; Sigma, St. Louis, MO, USA); ACTIN (Mouse; Proteintech, Wuhan, China); KDM4C (Rabbit; Sangon Biotech). Subsequently, the expression levels were measured by ECL reagents (Tanon, Shanghai, China) using Chemiluminescent Imaging System (Tanon). ACTIN or H3 were used as endogenous loading control.

### 4.6. Vector Construction and Virus Infection

To investigate the function of KDM4C, the Lentiviral overexpression plasmids of pig KDM4C was determined by NovoRec plus One step PCR Cloning Kit (Novoprotein, China). The genes were PCR-amplified from piPSCs and then subcloned into EF1-MCS-FLAG-T2A-puromycin Lentiviral vector [24]. In the Lentiviral vector, FLAG and KDM4C were fusion proteins, so FLAG could indicate the expression of KDM4C. Moreover, EF1-MCS-FLAG-T2A-puromycin Lentiviral vector was as a control to exclude the function of vector and transduction. 

Lentiviral vector was transfected into HEK293T cells using 1 mg/mL PEI (PolyScience, Niles, IL, USA) according to the manufacturer’s instruction. Then, the medium containing viral particles was obtained from each individual transfection and filtered through a 0.45 µm filter (Millipore, Burlington, MA, USA). For lentiviral infection, piPSCs were cultured on a gelatin-coated 6-well plate with 1 × 10^4^ cells per well. After 24 h, an equal ratio of viral particles was mixed and used to infect piPSCs with 4 µg/mL polybrene for 12 h. The infected piPSCs (simultaneously expressing FLAG and KDM4C, named as KDM4C-piPSCs) were then plated on feeder-coated 6-well plate after 12 h and cultured by the piPSCs induction medium with 4 µg/mL DOX for 5–7 days. Moreover, the piPSCs only expressing FLAG were used as a control (CON-piPSCs).

### 4.7. KDM4C Inhibitor Treatment

In order to verify the function of KDM4C in the CON- and E-piPSCs, the KDM4C inhibitor ML324 (MCE, USA) was added into the culture system of piPSCs. We chose different concentrations of ML324 (0, 2, 4 and 8 μM) and detected the change in the piPSCs after one passage. The ML324 was diluted with DMSO, and 0 μM was used as a DMSO control. We also added a mock control without adding DMSO. Moreover, the proliferation ability of the cells was detected by Cell-Light EdU Apollo643 in vitro Kit (RIB BIO, Guangzhou, China) according to the manufacturer’s instructions.

### 4.8. Immunoprecipitates–MS Analysis

To further understand the molecular mechanism of KDM4C, we applied immunoprecipitates–MS to directly investigate the proteins related to KDM4C. In the KDM4C-piPSCs, KDM4C and FLAG were fusion proteins, so anti-FLAG magnetic beads could be enriched proteins recruited by KDM4C. Moreover, FLAG-piPSCs were used as the control so that the effect of FLAG was excluded. The immunoprecipitate steps were described in the previous studies [5]. In brief, the cells were dissociated and pelleted, and the cytoplasm was removed by incubation in hypotonic buffer. Nuclei were pelleted, washed and permeabilized with 0.1% n-Octyl-β-D-glucopyranoside. Nuclei were digested with MNase at a concentration of 2 U/μL at 37 °C for 5 min. Nucleosomes with associated proteins were extracted in IP buffer, incubated with anti-FLAG magnetic beads overnight at 4 °C, washed five times with IP buffer and eluted with FLAG peptide at 150 ng/mL. Immunoprecipitates were subjected to SDS-PAGE and probed with indicated antibodies or detected by silver staining according to the manufacturer’s protocol. Then, the gel pieces were sent to PTM Biolab Technology Corporation (Wuhan, China). The resulting immunoprecipitate–MS data were processed using Proteome Discoverer 1.3. The KEGG analysis of proteins recruited by FLAG was plotted by http://www.bioinformatics.com.cn (accessed on 1 July 2021), an online platform for data analysis and visualization.

### 4.9. Statistical Analyses

All experiments were repeated at least three times. All data were analyzed using SPSS 18.0 (SPSS Inc., Chicago, IL, USA) and were expressed as means ± standard errors (SEs). ANOVA with Tukey’s HSD post hoc test was applied to multigroup comparisons, whereas Student’s *t* test was used for the two-group comparisons. When the *p* value was less than 0.05, the data were considered statistically significant.

## 5. Conclusions

Taken together, ESRRB could regulate KDM4C, and KDM4C also facilitated the expression of CDX2. These observations hint at the role of KDM4C as a key intermediary connecting the pluripotency network to transcriptional effectors in the iPSCs and TLSCs.

## Figures and Tables

**Table 1:** The primers used in this experiment.

Primer Name	Forward Sequence	Reverse Sequence
endo-ESRRB	GACGGGCAAGTTGCTGCTGACG	CGGTCCATCCATTTGTCTGTCC
endo-SOX2	ATGTCCCAGCACTACCAGAGCG	CTTACTCTCCTCCCATTTCCCTCT
CDX2	TGTGCGAGTGGATGCGGAAG	CTCCGAATGGTGATGTAGCGACTG
KRT8	TCAGATTTCCGACACCTCCG	AATCTCCGTCTTCGTGCGAC
TEAD4	CGCCTCAGCCTTCCACAATA	CGGCTGGACAGTGTAGGTTT
SMYD2	GCCAGGAAAGAAGGATTGTCCA	CCGTCAGCCTTACAGTCTCT
SETD2	CAGTTCATCGTCCAGTGCCT	AGGTCCTCCGGGTTCTTACA
NSD2	GGGATCTGGTGTGGTCCAA	GATACTGGCGGGCACTCTTT
KDM2A	AAGCCAGGTCAGGACAATCG	ACTGAGGTCGAGTCGAGACA
KDM2B	AGTGCTCCATCTGCAACGAA	CCACGCTTTTGCTTGTAGGC
KDM4A	CCTGGAAGAGGACTGCTGTTTATG	GGGACTTCTTTCTGCGATGTTG
KDM4B	CCTGCGGTGGATTGATTACGG	GTGAGGTCTTTGCCCTGCTTC
KDM4C	ATTCCAGCACCGATTCAGCA	GCTGCCTGAACTCCTTCACT

## Data Availability

Not applicable.

## Supplementary Materials

The following supporting information can be downloaded at: https://www.mdpi.com/article/10.3390/ijms23147586/s1.
