# Supplementary material for: KDM4C Contributes to Trophoblast-like Stem Cell Conversion from Porcine-Induced Pluripotent Stem Cells (piPSCs) via Regulating CDX2
Source: Int J Mol Sci. 2022 Jul 8;23(14):7586. doi: 10.3390/ijms23147586 (PMC9323581; doi:10.3390/ijms23147586)
Supplement: Supplementary file 1 [file ijms-23-07586-s001.zip › ijms-1799518-supplementary.pdf]

Supplementary Figures:

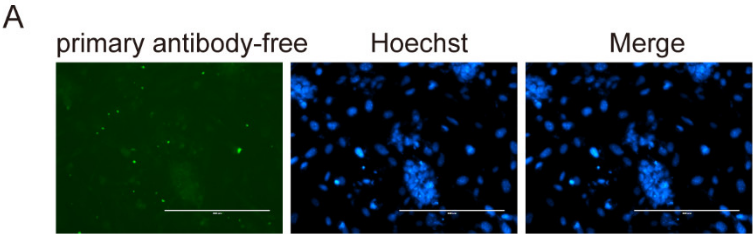

Figure S1. Immunofluorescence staining of no primary antibody (anti-Rabbit second antibody) control in the CON-piPSCs.

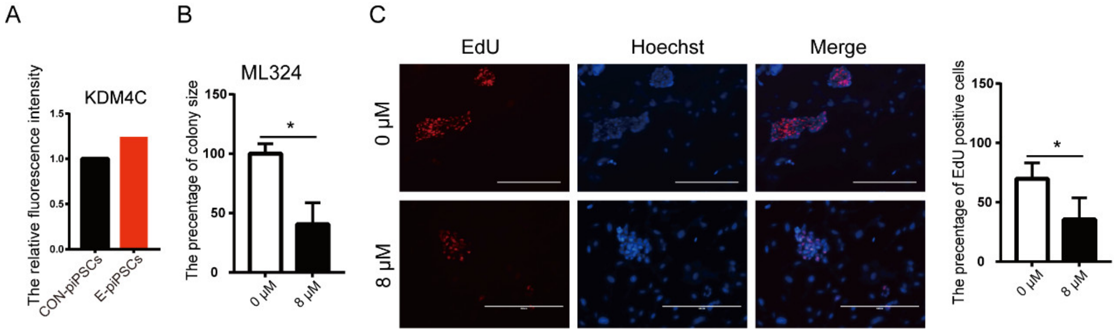

Figure S2. EdU staining analysis of CON-piPSCs after 8 μM supplement. (**A**) the quantification analysis of immunofluorescence staining of KDM4C in CON- and E-piPSCs. (**B**) The quantification analysis of colony size with adding 8 μM ML324 in CON-piPSCs; * represents $p < 0.05$. (**C**) EdU staining result and qualities analysis of the CON-piPSCs with adding 8 μM ML324; The scale bar represents 200 μm.

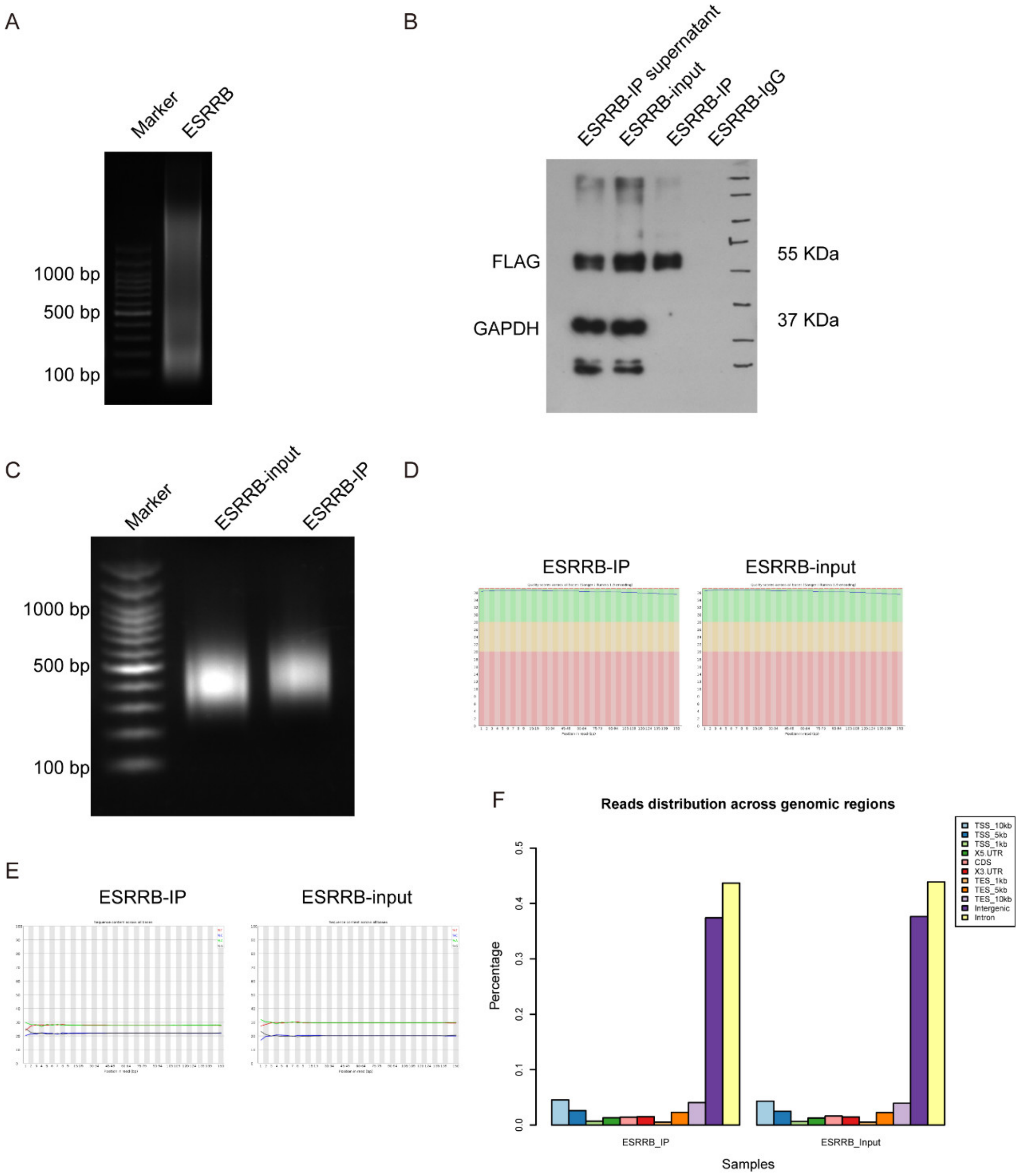

Figure S3. ChIP-seq quality inspection of E-piPSCs. (**A**) DNA ultrasonic results of E-piPSCs; the DNA fragments are distributed at 100–1000bp. (**B**) IP result of GAPDH and FLAG antibodies; the FLAG represents the expression of ESRRB. (**C**) the DNA database creation report. (**D**) Base mass distribution of ESRRB-FLAG ChIP-seq results. (**E**) Base balance of ESRRB-FLAG ChIP-seq results. (**F**) Histogram of the distribution of reads in different regions of the reference genome.

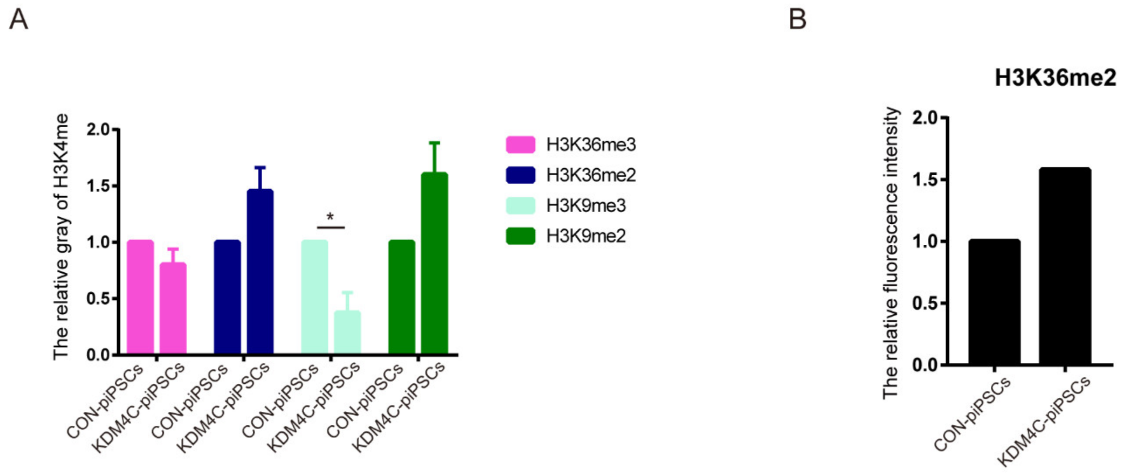

Figure S4. Quantification analysis of histone methylation in the CON- and KDM4C-piPSCs. (**A**) Quantification analysis of H3K36me3/2 and H3K9me3/2 of western blot in the CON- and KDM4C-piPSCs; (**B**) Quantification analysis of H3K36me2 immunofluorescence staining in the CON- and KDM4C-piPSCs.
